# Theophylline-encapsulated Nile Tilapia fish scale-based collagen nanoparticles effectively target the lungs of male Sprague–Dawley rats

**DOI:** 10.1038/s41598-022-08880-z

**Published:** 2022-03-22

**Authors:** Mohammed Moustapha Anwar, Manal Aly Shalaby, Hesham Saeed, Haitham Mohammed Mostafa, Dalia Galal Hamouda, Howaida Nounou

**Affiliations:** 1 Department of Biotechnology, Institute of Graduate Studies and Research (IGSR), Alexandria University, Alexandria, Egypt; 2 Department of Medical Biotechnology, Institute of Genetic Engineering, City of Scientific Research and Technological Applications, Alexandria, Egypt; 3 Centre of Excellence for Drug Preclinical Studies (CE-DPS) Pharmaceutical and Fermentation Industry Development Centre, City of Scientific Research and Technological Applications, New Borg El Arab, Alexandria, Egypt; 4 Department of Medical Biochemistry, Faculty of Medicine, Alexandria University, Alexandria, Egypt

**Keywords:** Nanoscience and technology, Nanoscale materials, Nanotoxicology

## Abstract

Nile Tilapia fish scale collagen has high biodegradability, excellent biocompatibility, and low antigenicity. We assessed both the encapsulation efficiency of theophylline into Nile Tilapia fish scale-based collagen nanoparticles and their stability as a pulmonary drug delivery system in male Sprague–Dawley rats. The present study has demonstrated the successful encapsulation of theophylline into the synthesised nanoparticles as shown by spectrophotometric analysis, light microscope, scanning electron microscope, transmission electron microscope, and dynamic light scattering. The antibacterial activity of the nanoparticles improves with increasing their concentrations. Intratracheal treatment of rats using theophylline-encapsulated nanoparticles reduced the levels of creatinine, alanine transaminase, and aspartate transaminase, compared to the control group. Nevertheless, nanoparticles combined with theophylline exhibited no effects on cholesterol and triglycerides levels. Histopathological examination revealed typical uniform and diffuse thickening of the alveolar walls with capillary oedema in treated rats. We concluded that the synthesised collagen nanoparticles appropriately target the lungs of male Sprague–Dawley rats when delivered via a nebuliser, showing good tolerability to lung cells. However, dose ratio of collagen nanoparticles to theophylline needs further evaluation. The nanoprecipitation method may be optimised to involve poorly water-soluble inhaled drugs, and avoid the drawbacks of traditional drug delivery.

## Introduction

Pharmaceutical compounds are characterised by unstable and rapid degradation profiles^1^. Nanoparticles are well known as drug delivery systems with a significant ability to control the particle shape, size, and charges^2^. Consequently, they are designed to achieve site-specific delivery and to improve the solubility and stability of drugs in different routes of administration, including oral, intravenous, and inhalation^3^. Collagen-based nanoparticles are interesting drug delivery candidates for the uptake of different drugs (e.g., retinol, theophylline, lidocaine, and tretinoin) and controlled drug release strategies due to their small size, large surface area, absorption capacity, and ease-of-adjustment^4–7^. Although collagen is used as a drug vehicle, the preparation of pure type-1 collagen requires the need to identify novel sources for affordable collagen extraction because the conventional methods are tedious and expensive^8,9^.

Collagen extracted from fish scales is an effective carrier and scaffold compared to bovine collagen. For instance, Nile Tilapia fish scale collagen (*Oreochromis niloticus*) is highly biodegradable and possesses cell growth potential, excellent biocompatibility, and low antigenicity^10^. Marine collagen has low molecular weight, lacks religious restraints, and carries no risk of transmitting diseases compared to mammalian-based collagen. It is biocompatible, cost-effective, easily absorbed into the bloodstream, and highly bioavailable compared to porcine or bovine collagen^11–13^. Fish scale-based collagen is sustainable and affordable, possesses appropriate water absorption and retention properties, making it suitable for therapeutic applications^14–16^. In human facial skin, Tilapia fish scale collagen effectively penetrated the stratum corneum into the epidermis and dermis, activating fibroblasts and accelerating collagen synthesis, and ultimately improving skin quality^17^.

Pulmonary drug delivery encounters various challenges to transport bronchodilators, including drug deposition, rapid clearance, and drug instability^18–20^. Theophylline—a hydrophilic drug—has a narrow therapeutic range with adverse effects at higher concentrations, making it the third-line bronchodilator for the treatment of asthma^21–23^. Furthermore, the encapsulation of theophylline for drug delivery with high loading efficiency remains a challenge, primarily due to its leakage to the exterior aqueous phase during the development of nanoparticles^24^. Generally, nanoparticles provide target-specific binding, improve the bioavailability of the loaded drugs and increase their half-life, and reduce their systemic toxicity^25,26^. Therefore, we aimed to evaluate the encapsulation efficiency of theophylline into Nile Tilapia fish scale-based collagen nanoparticles and their stability in male Sprague–Dawley rats. We hypothesised that targeting lung tissues of male Sprague–Dawley rats using Nile Tilapia fish scale-based collagen nanoparticles loaded with theophylline, may improve the targeted efficacy of theophylline and its systemic monitoring.

## Results

**Figure 1:**
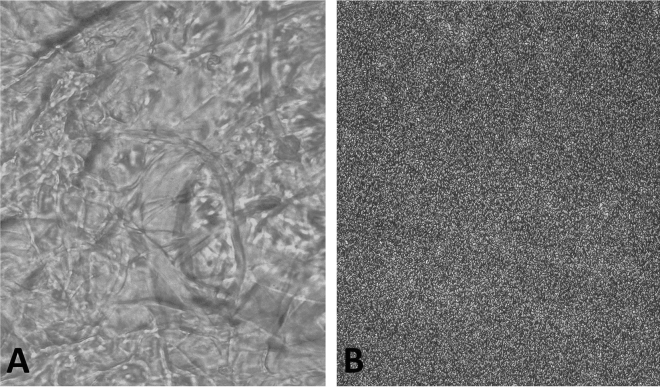
Light microscope (20X) images of collagen fibers extracted from Nile Tilapia fish scales (**A**) and aggregates of Nile Tilapia fish scale-based collagen nanoparticles (**B**).

In the present study, Nile Tilapia fish scales produced about 40% of the readily soluble collagen in dilute acetic acid, accomplished via primary decalcification step that exposed the bundles of collagen fibrils to enable their dissolution by direct solute–solvent interaction. Non-solvent precipitation was the primary method used for the preparation of Nile Tilapia fish scale-based collagen nanoparticles. By mixing the aqueous solution with ethanol and acetone, the protein became supersaturated, the protein nuclei formed while being surrounded by the free condensed protein, creating protein nanoparticles. Increased turbidity of the collagen solution after the stepwise addition of acetone/ethanol indicated the formation of nanoparticles. Turbidity of collagen solution demonstrated that the optimum temperature was 37 °C, while the optimum pH was 5. Light microscope confirmed the preparation of Nile Tilapia fish scale-based collagen nanoparticles by the desolvation method (Fig. 1).

### Characterisation of Nile Tilapia fish scale-based collagen nanoparticles

**Figure 2:**
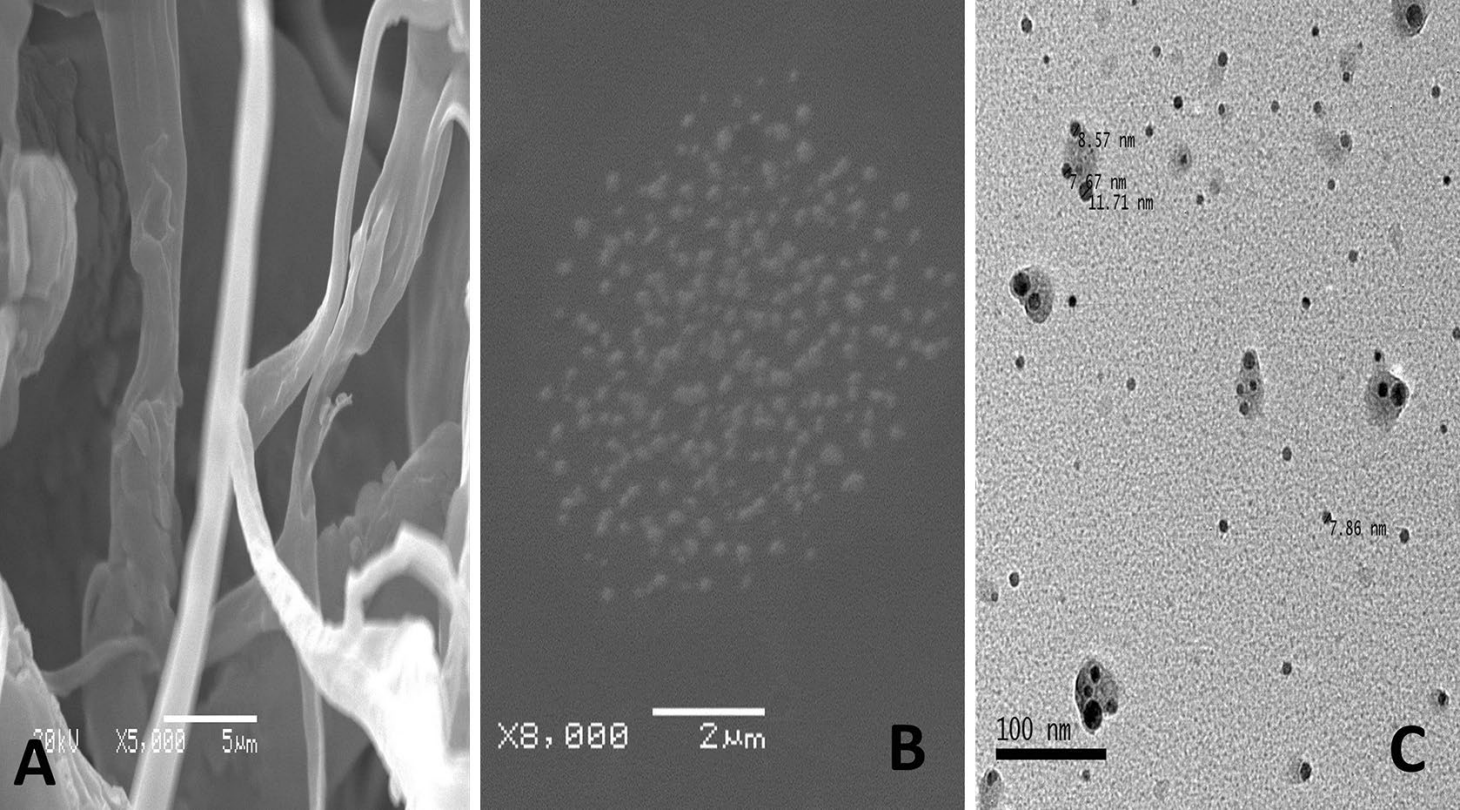
A scanning electron microscope (SEM) (5X) image showing Nile Tilapia fish scale-based collagen fibers (**A**), Nile Tilapia fish scale-based collagen nanoparticles showing smooth spherical shape by SEM (5X) (**B**), Nile Tilapia fish scale-based collagen nanoparticles surrounded by corona using transmission electron microscope (TEM) (8X) (**C**).

**Figure 3:**
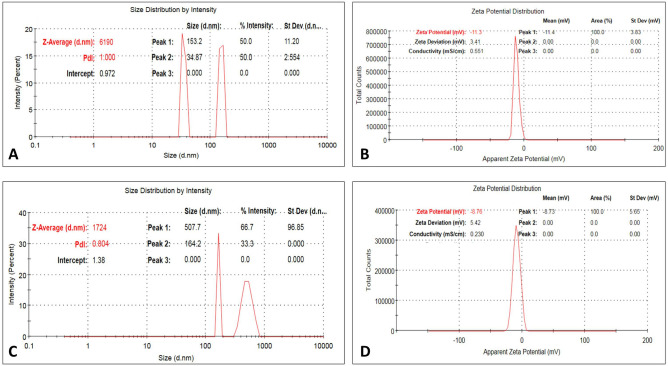
Nile Tilapia fish scale-based collagen nanoparticles prepared by ethanol desolvation showing size distribution by intensity (**A**) and zeta (ζ) potential distribution (**B**). Nile Tilapia fish scale-based collagen nanoparticles prepared by acetone desolvation showing size distribution by intensity (**C**) and ζ potential distribution (**D**).

The transmission electron microscopy (TEM) (8X) images illustrate spherical collagen nanoparticles with a size of 7.67, 8.57, and 11.71 nm, where the surface of most of the Nile Tilapia fish scale-based collagen nanoparticles is decorated by a protein shell. The zeta (ζ) potential distribution of Nile Tilapia fish scale-based collagen nanoparticles falls in a narrow range, which provides a high surface area, a better catalytic activity, and a high dispersion capacity^27^. The corresponding average ζ value is − 11.3 mV and – 16.1 mV of Nile Tilapia fish scale-based collagen nanoparticles prepared by ethanol and acetone, respectively. Figure 2 shows the scanning electron microscope (SEM) (5X) and TEM (8X) images of Nile Tilapia fish scale-based collagen fibers and nanoparticles with different sizes of spherical structures and smooth surfaces. The size distribution by dynamic light scattering (DLS) revealed that Nile Tilapia fish scale-based collagen nanoparticles are polydispersed in nature, with an average diameter of approximately 34–153 nm and 433 nm using ethanol and acetone, respectively (Fig. 3).

### Cellular uptake of Nile Tilapia fish scale-based collagen nanoparticles by peripheral blood mononuclear cells (PBMCs)

**Figure 4:**
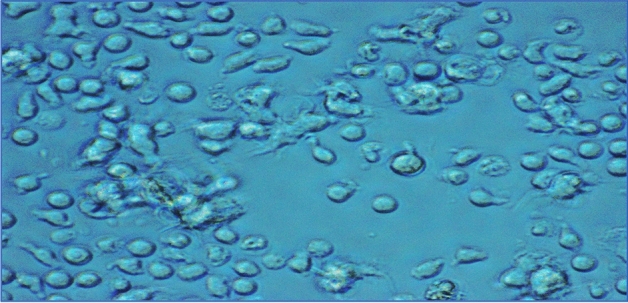
Light microscope (20X) image of the cellular uptake of Nile Tilapia fish scale-based collagen nanoparticles by peripheral blood mononuclear cells (PBMCs), showing cells with filopodia and lamellipodium.

Upon incubation, we observed aggregates of Nile Tilapia fish scale-based collagen nanoparticles in PBMCs under inverted light microscope. We also noticed that Nile Tilapia fish scale-based collagen nanoparticles initiated filopodia from both sides of the PBMCs, moving laterally through the lamellipodium, merging and moving into the cell body while cells continued to move forward (Fig. 4).

### Antibacterial activity of Nile Tilapia fish scale-based collagen nanoparticles

**Figure 5:**
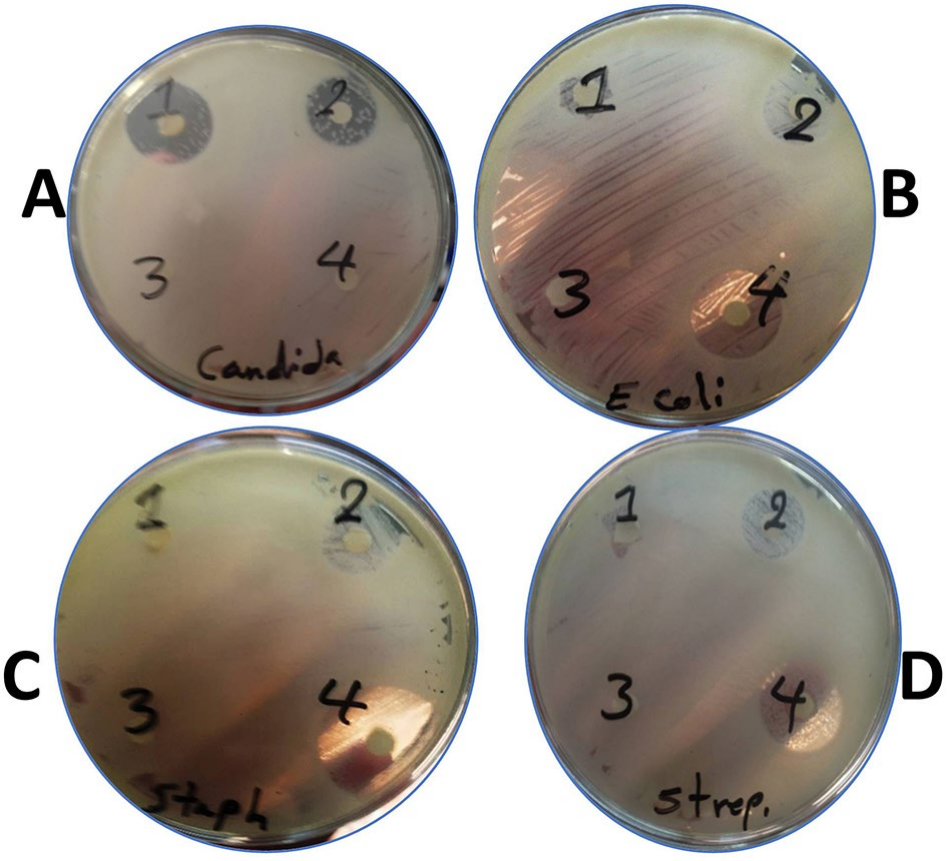
Antimicrobial activity of Nile Tilapia fish scale-based collagen nanoparticles prepared by ethanol showing clear zones of bacterial inhibition against *Candida albicans* (*C. albicans*) (**A**), *Escherichia coli* (*E. coli*) (**B**), *Staphylococcus aureus* (*S. aureus*) (**C**) *Streptococcus mutans* (*S. mutans*) (**D**) using 5 μg (1) and 10 μg (2). Antimicrobial activity of Nile Tilapia fish scale-based collagen nanoparticles prepared by acetone showing clear zones of bacterial inhibition against *S. aureus*, *S. mutans*, *E. coli,* and *C. albicans* using 5 μg (3) and 10 μg (4).

We investigated the antibacterial activities of Nile Tilapia fish scale-based collagen nanoparticles against *Staphylococcus aureus* (*S. aureus*), *Streptococcus mutans* (*S. mutans*), *Escherichia coli* (*E. coli*), and *Candida albicans* (*C. albicans*) by agar disk diffusion method. The Nile Tilapia fish scale-based collagen nanoparticles prepared by acetone were more active than those prepared by ethanol. We also found that the antibacterial activity improved by increasing the concentration of Nile Tilapia fish scale-based collagen nanoparticles (Fig. 5).

### Encapsulation of theophylline in Nile Tilapia fish scale-based collagen nanoparticles

**Figure 6:**
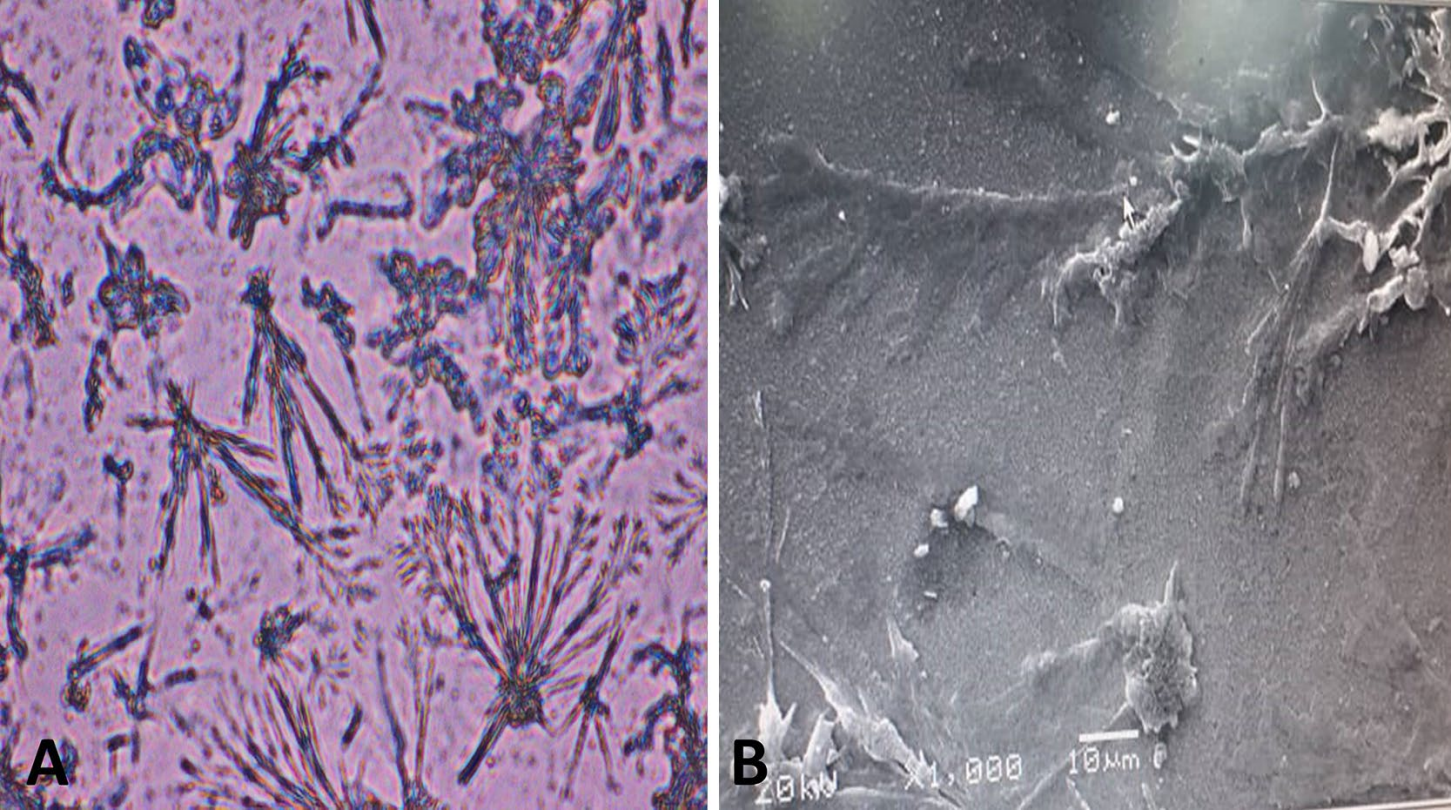
Attachment of Nile Tilapia fish scale-based collagen nanoparticles to theophylline showing the needle structure of theophylline as examined by light microscope (20X) (**A**) and scanning electron microscope (SEM) (5X) (**B**).

Spectrophotometric, light microscopy, TEM, and SEM analyses confirm the encapsulation of theophylline in Nile Tilapia fish scale-based collagen nanoparticles (Fig. 6). Spectrophotometric analysis demonstrated that theophylline remains attached to Nile Tilapia fish scale-based collagen nanoparticles even after washing 3 times. Scanning electron microscope analysis showed that anhydrous theophylline particles consist of primary crystallites attached together to create needle–shaped particles. Nile Tilapia fish scale-based collagen nanoparticles also formed porous and spherical agglomerates to which submicron theophylline particles are attached.

### Assessment of aspartate aminotransferase (AST), alanine aminotransferase (ALT), and creatinine levels in serum

**Figure 7:**
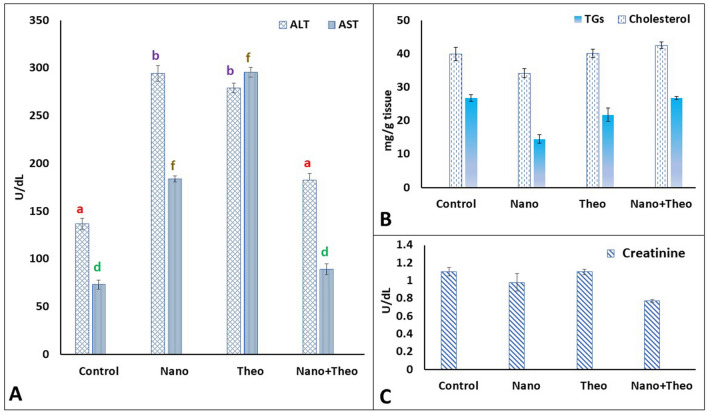
The effect of Nile Tilapia fish scale-based collagen nanoparticles and theophylline on aspartate aminotransferase (AST) and alanine aminotransferase (ALT) levels (**A**), cholesterol and triglycerides levels (**B**), and creatinine levels (**C**) in serum. Letters **a**, **b**, **d**, and **f** denote statistically significant difference between groups. All treatment groups had no significant effect on the levels of triglycerides, cholesterol, and creatinine. ALT, Alanine Aminotransferase; AST, Aspartate Aminotransferase; Nano = Nile Tilapia fish scale-based collagen nanoparticles; Theo = Theophylline; TGs, Triglycerides.

No mortality was observed in either treated male Sprague–Dawley rats or in the control group. Male Sprague–Dawley rats did not show abnormal behaviour for 7 days after treatment. Microscopical examination of the liver did not show any evidence of toxicity. Intratracheal treatment with Nile Tilapia fish scale-based collagen nanoparticles significantly increased levels of AST to 295.4 mg/dL (*P* < 0.0001) versus the control group (saline), while theophylline alone increased AST levels to 184 mg/dL (*P* < 0.0001). Particularly, theophylline-harbouring Nile Tilapia fish scale-based collagen nanoparticles reduced levels of AST to 136.6 mg/dL (*P* = 0.2), compared to the control group (Fig. 7). Nile Tilapia fish scale-based collagen nanoparticles upregulated levels of ALT significantly up to 278.9 mg/dL (*P* < 0.0001) versus the control group, whereas theophylline alone significantly increased the levels to 294.39 mg/dL (*P* < 0.0001). Nile Tilapia fish scale-based collagen nanoparticles loaded with theophylline reduced levels of ALT against the control group to 136.6 mg/dL (*P* = 0.01) (Fig. 7).

All treatments did not significantly change the levels of creatinine (*P* > 0.2) as compared to the control group. Treatment with Nile Tilapia fish scale-based collagen nanoparticles slightly decreased levels of creatinine to 0.98 mg/dL against the control group, while theophylline alone had no effect. A combination of theophylline and Nile Tilapia fish scale-based collagen nanoparticles reduced levels of creatinine to 0.77 mg/dL compared to the control group (Fig. 7).

### Assessment of cholesterol and triglycerides in liver homogenate

All treatments had no significant effects on the levels of either cholesterol or triglycerides significantly versus the control group (*P* > 0.2). Intratracheal administration of Nile Tilapia fish scale-based collagen nanoparticles reduced both levels of cholesterol and triglycerides to 34.2 and 14.5 mg/g tissue compared to the control group, respectively. In contrast, theophylline alone increased levels of cholesterol and triglycerides to 40.1 and 21.75 mg/g tissue with an increment of 0.25% for cholesterol and 45.62% decrement for triglycerides. In addition, treatment with Nile Tilapia fish scale-based collagen nanoparticles combined with theophylline exhibited no effect on levels of cholesterol and triglycerides compared to the control group (Fig. 7).

### Histopathological examination

**Figure 8:**
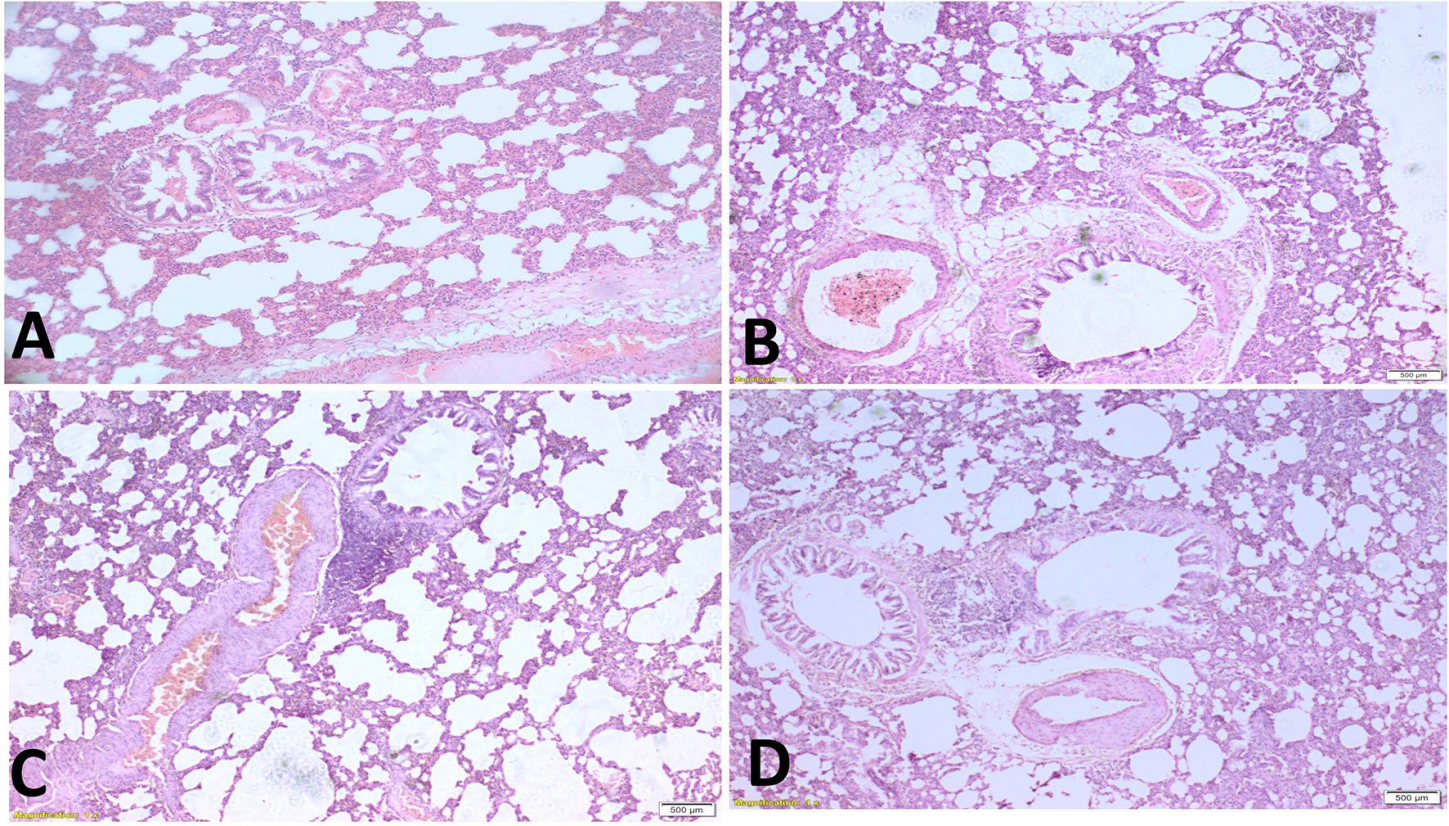
Histological evaluation of male Sprague–Dawley rats lung tissues stained with Haematoxylin and Eosin (H&E). Untreated lung tissues (**A**), lung tissues treated with Nile Tilapia fish scale-based collagen nanoparticles (**B**), lung tissues treated with theophylline (**C**), lung tissues treated with Nile Tilapia fish scale-based collagen nanoparticles loaded with theophylline (**D**).

The lungs of male Sprague–Dawley rat control group showed thin interstitial alveolar wall and capillary vessels (Fig. 8). All treatment groups had typical uniform and diffuse thickening of the alveolar walls with capillary congestion due to oedema. However, the lungs of theophylline-treated group had rare inflammatory cells. Nile Tilapia fish scale-based collagen nanoparticles-treated groups showed some degree of protein debris in the air spaces. Theophylline-encapsulated Nile Tilapia fish scale-based collagen nanoparticles exhibited infiltrating inflammatory cells, with little migration of lymphocytes.

## Discussion

We have found that theophylline-harbouring Nile Tilapia fish scale-based collagen nanoparticles are a stable and a safe drug delivery system. We hypothesised that targeting lung tissues using theophylline-encapsulated Nile Tilapia fish scale-based collagen nanoparticles and identifying their toxicity in-vivo, may enhance targeted inhalation therapy and systemic monitoring. One unexpected finding was the presence of infiltrating inflammatory cells accompanied by little migration of lymphocytes upon treatment with theophylline-encapsulated theophylline-harbouring Nile Tilapia fish scale-based collagen nanoparticles.

The nanoprecipitation method carried out in the present study successfully led to the instantaneous formation of Nile Tilapia fish scale-based collagen nanoparticles^28^. Hornig et al., reported that the nanoprecipitation technique is simple, easy, versatile, and broadly applicable without any additives to produce well-defined nanoparticles^29^. In the current study, SEM (5X) images showed distinguished collagen fibers that turn into spherical accumulations with smooth surface, highlighting the formation of Nile Tilapia fish scale-based collagen nanoparticles. These results agree with Elzoghby et al., in which the nanoprecipitation technique helped in the supersaturation of collagen with the formation of collagen nuclei surrounded by free condensed collagen units^30^. In accordance with Salatin et al., the nanoprecipitation method created small-sized collagen nanoparticles characterised by narrow size distribution (7.67, 8.57, and 11.71 nm)^31^.

Transmission electron microscopy analysis showed an exterior protein shell that decorated the surface of most of the Nile Tilapia fish scale-based collagen nanoparticles, forming what is known as ‘protein’ or ‘bio corona.’ This finding agrees with Gunawan et al., and Bai et al., who highlighted that the presence of protein corona–cell interactions might have either beneficial or adverse biological effects on nanomedicine^32,33^. These effects include ease-of-targeted delivery, cell-specific uptake of therapeutic nanoparticles, vaccine development based on engineered nanoparticles as adjuvants, and reduction of the nanoparticles’ immunotoxicity^34^. Moreover, Di Marco et al., showed that the protein corona reduced adhesion and agglomeration of nanoparticles, which helps avoid recognition by the immune system or thrombosis, especially after injections^35^. Nevertheless, other reports showed that the protein corona elicited inflammatory responses, physiological and pathological changes, as well as unknown toxicity mechanisms^36–40^. In the present study, we have found infiltrating inflammatory cells with less migration of lymphocytes following treatment with theophylline-encapsulated collagen nanoparticles. The presence of lymphocytes was confirmed by observing protein debris in the air spaces caused by treatment with Nile Tilapia fish scale-based collagen nanoparticles (Fig. 8), which requires further investigation.

Size distribution and surface charge are key determinants of the stability of the biosynthesised collagen nanoparticles^41^. Shao et al., concluded that nanoparticles with positive ζ potential were significantly more cytotoxic than others with negative ζ potential, due to stronger interactions with the cells^42^. The current data obtained from DLS measurements illustrated that Nile Tilapia fish scale-based collagen nanoparticles are polydispersed in nature with an average ζ potential value of − 11.3 mV and − 16.1 mV using ethanol and acetone, respectively. Such negative values of ζ potential designate a negative net charge on the surface of the nanoparticles. Previous studies explained that higher negative ζ values of nanoparticles support their good colloidal nature, stability, and greater dispersity by creating an interparticle electrostatic repulsion that prevents aggregation and ultimately thrombosis in cases of intravenous injections^41,43^. In the present study, the calculated ζ potential had no effect on morphology, which confirms the spherical shape of collagen nanoparticles. We also noticed that the particle size of the synthesised Nile Tilapia fish scale-based collagen nanoparticles was equally distributed, showing better size distribution that was in accordance with DLS measurements.

The encapsulation of water-soluble drugs (e.g., theophylline) into nanoparticles is more difficult than hydrophobic drugs because the former have a higher affinity towards the outer aqueous phase, resulting in a decreased loading efficiency^44^. Comparisons of the present findings with other studies confirm that using water-miscible organic desolvating agents (i.e., acetone and ethanol) has facilitated the encapsulation of theophylline, allowing it to divide between water and organic phase^28,30^.

The size of nanoparticles regulate the pathway of endocytosis. For example, a maximum size of 200 nm is the limit for internalisation via the clathrin-mediated endocytosis, compared to caveolae-mediated internalisation that is specific to particles of 500 nm^45^. Perde-Schrepler et al., showed that nanoparticles of 5 nm were the most genotoxic in tumoural cell lines due to the production of reactive oxygen species (ROS), compared to larger nanoparticles^46^. In the current study, the size of the synthesised Nile Tilapia fish scale-based collagen nanoparticles was 7.67, 8.57, and 11.71 nm, which is markedly less than 200 nm and more than 5 nm, being a good candidate for drug delivery.

Studying nanomaterials with PBMCs is important since these cells interact with nanoparticles once introduced into the blood circulation^47^. In the current study, we have found agglomerates of Nile Tilapia fish scale-based collagen nanoparticles that are merged into PBMCs through which PBMCs initiated filopodia from both sides to move laterally across the lamellipodium. Specifically, lateral movement is essential to allow the structure to recognise any stimuli before its adhesion with other cells/substrates. Peripheral blood mononuclear cells use the dynamic filopodial protrusions to recognise the surrounding environment, presence of other cells, and check for proper directions and matrix^48^. The distribution and migratory properties of the synthesised Nile Tilapia fish scale-based collagen nanoparticles could enhance drug delivery^49^.

The present work confirms the correlation between particle size and the antimicrobial effects of nanoparticles as previously reported^50^. We have noticed that Nile Tilapia fish scale-based collagen nanoparticles were effective as an antibacterial against *E. coli*,* S. mutans*, *S. aureus*, and *C. albicans* using the agar disk diffusion method. Such results reveal that the Nile Tilapia fish scale-based collagen nanoparticles might be useful as a broad-spectrum antibacterial agent. Narayanan et al., reported that the antibacterial activity increased with decreasing particle size and increasing concentration of zinc oxide (ZnO) nanoparticles^50^.

We examined the encapsulation efficiency of theophylline into Nile Tilapia fish scale-based collagen nanoparticles, to get better insights about the role of collagen nanoparticles as a drug carrier. The encapsulation efficiency of theophylline into Nile Tilapia fish scale-based collagen nanoparticles was above 30%. For liposomes, Guimarães et al., recently reported that small size, polydispersity index, and a higher encapsulation efficiency (i.e., higher than 30%), are suitable characteristics for in-vivo application^51^. Data also reveal that theophylline usually exhibits a strong and a broad absorption peak at 275 nm^52^. In the current study, theophylline-encapsulated Nile Tilapia fish scale-based collagen nanoparticles showed a maximum absorption peak at 275 nm, even after three times washing, confirming the strong attachment of theophylline to the synthesised Nile Tilapia fish scale-based collagen nanoparticles. Moreover, light microscopy and high-resolution SEM (5X) images highlighted the attachment of Nile Tilapia fish scale-based collagen nanoparticles to the crystalline needle structure of theophylline.

Treatment of male Sprague–Dawley rats with Nile Tilapia fish scale-based collagen nanoparticles alone and theophylline-loaded Nile Tilapia fish scale-based collagen nanoparticles, have not induced mortality or significant signs of toxicity for 7 days postinjection, compared to the control group. Moreover, Nile Tilapia fish scale-based collagen nanoparticles have not caused significant changes in all the tested biochemical parameters (i.e., ALT, AST, creatinine, cholesterol, and triglycerides). On the one hand, Nile Tilapia fish scale-based collagen nanoparticles-treated male Sprague–Dawley rats have experienced elevated levels of AST and ALT; however, they have survived throughout the experiment, indicating that Nile Tilapia fish scale-based collagen nanoparticles are safe. On the other hand, theophylline-harbouring Nile Tilapia fish scale-based collagen nanoparticles have reduced both levels of AST and ALT compared to Nile Tilapia fish scale-based collagen nanoparticles alone. Such reduction may be attributed to the surface changes of Nile Tilapia fish scale-based collagen nanoparticles after coupling with theophylline, showing that the metabolism was not disturbed by using Nile Tilapia fish scale-based collagen nanoparticles as a targeted drug delivery system.

While the liver and kidneys are the targets of nanoparticles in rats, administration of Nile Tilapia fish scale-based collagen nanoparticles have not demonstrated detectable systemic toxicity, changes in either the overall health of the rats, or the profile of the developed collagen nanoparticles. Given the protein corona that surrounds the collagen nanoparticles as shown by SEM (5X) analysis, Nile Tilapia fish scale-based collagen nanoparticle could experience various changes while moving from one compartment or fluid to another, due to the presence of the protein corona. In addition, several studies have outlined that proteins in the corona may have lost or gained functions, increase inflammatory response, have perturbed structures and aggregation propensity, and possess complexes of apolipoproteins with phospholipids, cholesterol, and triglycerides^53^. However, theophylline-encapsulated Nile Tilapia fish scale-based collagen nanoparticles in the present experiment had no effect on cholesterol or triglyceride levels versus treatment with either Nile Tilapia fish scale-based collagen nanoparticles or theophylline alone. Additionally, Nile Tilapia fish scale-based collagen nanoparticles proved to be nontoxic upon detection using serum creatinine that is specific to renal functions, where renal damage elevates serum creatinine in mammals^54^.

Histopathological examination in the current work revealed that the intratracheal delivery of theophylline inhibited the infiltration of inflammatory cells beneath the epithelium and reduced the epithelial damage in male Sprague–Dawley rats. We have not observed inflammation, although the combination of theophylline with Nile Tilapia fish scale-based collagen nanoparticles penetrated the lung tissues through the intratracheal route, causing oedema of bronchioles and blood vessels.

### Strengths and limitations

A key strength of the present study was the successful encapsulation of theophylline into Nile Tilapia fish scale-based collagen nanoparticles, exhibiting sustained release of theophylline that increases its half-life, reduces the frequency of dosing, improves its bioavailability, and ameliorates patient adherence and compliance accordingly. Moreover, loading of theophylline as a hydrophilic drug on Nile Tilapia fish scale-based collagen nanoparticles would open novel horizons to investigate the possibility of delivering other unstable inhaled medications.

Contrarily, this study has several limitations. First, we have not characterised Nile Tilapia fish scale-based collagen nanoparticles by using a human bronchial epithelial cell line. Second, we have not assessed the in-vitro drug release of theophylline upon loading on Nile Tilapia fish scale-based collagen nanoparticles, its deposition properties upon nebulisation, and its transport across the airway epithelium of human cell lines. Third, the experimental male Sprague–Dawley rats were healthy and were not induced to trigger asthma; we have not also tested whether Nile Tilapia fish scale-based collagen nanoparticles have adverse effects on other organs, such as the heart and stomach.

## Conclusions and future directions

We concluded that despite their small size, the synthesised Nile Tilapia fish scale-based collagen nanoparticles are able to target the appropriate area of the lungs when delivered using a nebuliser and are well tolerated by airway cells. We also confirm that the nanoprecipitation method could be optimised to incorporate inhaled drugs with poor aqueous solubility and help overcome the limitations of conventional drug delivery, including drug stability. Further research is necessary to explain the formation of a protein corona upon contact with plasma or blood and to identify its benefits and drawbacks. Dose ratio of Nile Tilapia fish scale-based collagen nanoparticles to theophylline requires further adjustments for a successful drug delivery.

## Materials and methods

### Isolation of collagen from Nile Tilapia fish scales

Collagen was isolated from the scales of Nile Tilapia fish scales collected by sea catch, washed thoroughly with tap water, and lyophilised^55^. We previously developed, validated, and characterised the type 1 collagen used in the current research, using different physicochemical characterisation such as sodium dodecyl sulfate–polyacrylamide gel electrophoresis (SDS-PAGE), Fourier-transform infrared (FTIR) spectroscopy, SEM, and TEM analyses^55^. To remove the noncollagenous proteins and pigments, the lyophilised collagen was treated with 0.1 N NaOH for 3 days, washed using distilled water, dried, and stored at 80 °C. We extracted the dried matter using 0.5 M acetic acid for 3 days followed by centrifugation at 50,000 *g* for 1 h, and we pooled the supernatant by adding sodium chloride (NaCl) gradually to a final concentration of 0.9 M. The pellet was washed, reprecipitated by distilled water 3 times to remove the salt, suspended in 0.5 M acetic acid, freeze-dried, grounded into a powder by a personal-sized mill, and sieved with a 0.15-mm sieve mesh.

### Preparation and purification of collagen-theophylline nanoparticles

We prepared the Nile Tilapia fish scale-based collagen nanoparticles by the ‘nanoprecipitation method’ by mixing the aqueous solution (i.e., water) with the drop-wise addition of 50 mL absolute ethanol or acetone as desolvating agents. Glutaraldehyde (500 µL) was added as a cross-linking material to form the collagen nanoparticles. Nile Tilapia fish scale-based collagen nanoparticles were centrifuged at 20,000 rpm for 30 min and filtered using a 0.22-μm hydrophilic polyvinylidene fluoride (PVDF) membrane (Merck Millipore, Billerica, MA, USA)^28,56^. For drug loading, 0.5 g theophylline was added step-wise to an aqueous solution of 0.5 g Nile Tilapia fish scale-based collagen nanoparticles with continuous stirring. Then, glutaraldehyde was added with stirring to induce particle crosslinking. The solution of theophylline-encapsulated Nile Tilapia fish scale-based collagen nanoparticles was centrifuged, lyophilised, and stored for further applications.

Light microscope (20X) (B & B Microscopes, Olympus, cat. no. CKX31) was used to confirm the formation of Nile Tilapia fish scale-based collagen nanoparticles^57^. We evaluated the encapsulation efficiency (EE) of theophylline according to Charehsaz et al., 2014 by spectrophotometry (Spectrostar^Nano^, BMG Labtech)^58^ and calculated its percentage using the below formula^59^:

$${\text{EE}}\% = ({\text{W}}_{{\text{p}}} /{\text{W}}_{{\text{t}}} ) \times 100\%$$

W_P_: the total amount of the encapsulated purified theophylline, W_t_: the total quantity of the encapsulated theophylline during the preparation.

### Characterisation of Nile Tilapia fish scale-based collagen nanoparticles

Light microscope, SEM, TEM, and DLS analyses were performed to characterise the formed Nile Tilapia fish scale-based collagen nanoparticles^57,60,61^. We cut the produced Nile Tilapia fish scale-based collagen nanoparticles into sections to determine their size by light microscope. We also used a punch to chop the Nile Tilapia fish scale-based collagen nanoparticles and fixed them to a carbon adhesive stub for analysis by a Tabletop SEM (Hitachi High-Technologies Corp., Japan) (5X) operated at 15 kV. Furthermore, TEM (8X) (TALOS Instrument, Thermo Fischer Scientific, USA) was used to examine the morphology and particle size distribution of Nile Tilapia fish scale-based collagen nanoparticles. For a better dispersion, the solution of Nile Tilapia fish scale-based collagen nanoparticles was sonicated for 5 min to prepare the TEM sample. Then, we placed one drop of the TEM sample on a 200–300-mesh carbon-coated copper grid and left in the air to dry for imaging.

We used the DLS technique to determine the size distribution and ζ potential of the produced Nile Tilapia fish scale-based collagen nanoparticles. Measurements were carried out on a Malvern Zetasizer (Malvern Instruments Corp., Malvern, United Kingdom) in solutions of pH = 5. All samples were diluted with Millipore-filtered (MF-Millipore™ Membrane Filters) deionised water to an appropriate scattering intensity.

### Evaluation of the antimicrobial effect of Nile Tilapia fish scale-based collagen nanoparticles

The extracted collagen has been previously tested positive for antimicrobial activity alongside control streptomycin antibiotic^55^. The antimicrobial effect of Nile Tilapia fish scale-based collagen nanoparticles was determined by in-vitro agar disk diffusion method against *S. aureus* (ATCC 25923), *S.* *mutans* (ATCC 5175), *E.* *coli* (ATCC 25922), and *C. albicans* (ATCC 10231)^50,62^. Standard bacterial cultures were obtained from the Genetic Engineering and Biotechnological Research Institute (GEBRI), City of Scientific Research and Technology Applications (SRTA-CITY), New Borg El Arab, Alexandria, Egypt.

The Nile Tilapia fish scale-based collagen nanoparticles prepared by ethanol/acetone were washed twice with double-distilled water (ddH_2_O), then injected on sterilised discs after microbial inocula were distributed on LB agar plates. After 18 h of incubation at 37 °C, the inhibition zones on the triplicate plates were measured. To calculate the minimum inhibitory concentration (MIC), distances were measured and examined separately, with the average results using the triplicate plates. An Accurate assessment of bacterial susceptibility to Nile Tilapia fish scale-based collagen nanoparticles is crucial to prevent the spread of bacterial infections^63^.

### Cellular reuptake of Nile Tilapia fish scale-based collagen nanoparticles by peripheral blood mononuclear cells (PBMCs)

The Ficoll-Paque method was used for the rapid isolation of PBMCs from the peripheral blood^64^. After 60% confluence of cell culture, cells are exposed to the sterile filtered Nile Tilapia fish scale-based collagen nanoparticles for 24 h, whereas nonexposed cells were considered as a negative control. Cell viability was observed under light microscopy. To study the Nile Tilapia fish scale-based collagen nanoparticles-lymphocyte cell interaction, lymphocytes were incubated for different periods in the presence of Nile Tilapia fish scale-based collagen nanoparticles’ solution at different concentrations with various particle sizes.

### In-vivo cytotoxicity assessment of Nile Tilapia fish scale-based collagen nanoparticles

All male Sprague–Dawley rat experiments followed the Animal Research: Reporting In-Vivo Experiments (ARRIVE) guidelines and were executed according to the U.K. Animals (Scientific Procedures) Act, 1986 and national or institutional guidelines for the care and use of animals^65^. This study was conducted in accordance with the institutional guidelines for the care and use of laboratory animals established by the Animal Ethics Committee of Alexandria University initiated in July 2005. All animal procedures and facilities related to the purpose of the research were reviewed and approved by the institutional review board (IRB) at the faculty of medicine here at Alexandria University. A total of 24 male Sprague–Dawley rats (250–280 g) were served with standard food pellets and tap water ad libitum and kept in standard conditions (a 12-h light/dark cycle, 25ºC, and relative humidity of 20%). We categorised the rats into four different groups (n = 10) as in Table 1. Rats were allowed to fast for 12 h after the last dose of treatment. Male Sprague–Dawley rats were anesthetised and sacrificed by decapitation, and we collected the blood in ethylenediamine tetraacetic acid- (EDTA) containing tubes. To separate blood plasma, the sample tubes were centrifuged at 1500 rpm for 10 min at 4 °C. Processing of lung tissue was performed according to Nounou et al.^66^.

**Table 1:** Categorisation of treatment groups

Group number	Assigned treatment for 7 days
I (Control group)	Intratracheal saline
II	40 mg/kg collagen nanoparticles
III	40 mg/kg theophylline
VI	40 mg/kg of collagen nanoparticles loaded with theophylline

### Assessment of AST, ALT, and creatinine levels in serum

The kinetic method was carried out using the AST and ALT kits (Spectrum, Schiffgraben, Hannover, Germany) according to the manufacturer's instructions^67^. Creatinine level was measured by the buffered Kinetic jaffé reaction without deproteinisation, using the creatinine determination kit (Spectrum, Schiffgraben, Hannover, Germany) according to the manufacturer's instructions. Assessment was carried out in four different groups as in Table 1.

### Assessment of cholesterol and triglyceride levels in liver homogenate

Total cholesterol and triglyceride levels were measured by the CHOD-PAP-enzymatic colorimetric method and the GPO-PAP-enzymatic colorimetric method using the cholesterol and triglycerides kits (Spectrum, Schiffgraben, Hannover, Germany), respectively, according to the manufacturer's manual. Assessment was carried out in four different groups as in Table 1.

### Standard histology

We prepared the histological sections from lung tissue specimens (i.e., obtained by sharp dissection, fixed by 10% formalin, and embedded in paraffin for serial sectioning). Representative sections underwent staining by Haematoxylin and Eosin (H&E)^43,52^.

### Statistical analysis

All results were expressed as mean ± standard error of the mean (s.e.m.). Multiple comparisons were carried out using one-way analysis of variance (ANOVA) followed by the posthoc test and the P-value < 0.05 was accepted as the level of significance. All statistical tests and Figures were carried out using GRAPHPAD PRISM version 6 (GRAPHPAD software, USA).

## Data Availability

The datasets generated during and/or analysed during the current study are included in this article.
